# Screening primary carnitine deficiency in 10 million Chinese newborns: a systematic review and meta-analysis

**DOI:** 10.1186/s13023-024-03267-x

**Published:** 2024-07-03

**Authors:** Jinfu Zhou, Guilin Li, Yinglin Zeng, Xiaolong Qiu, Peiran Zhao, Ting Huang, Xi Wang, Jinying Luo, Na Lin, Liangpu Xu

**Affiliations:** 1 Medical Genetic Diagnosis and Therapy Center, Fujian Key Laboratory for Prenatal Diagnosis and Birth Defect, Fujian Maternity and Child Hospital College of Clinical Medicine for Obstetrics & Gynecology and Pediatrics, Fujian Medical University, Fuzhou, 350001 Fujian Province China; 2 Department of Preventive Medicine, School of Public Health, Fujian Medical University, Fuzhou, 350122 Fujian Province China; 3 Department of Clinical Laboratory, Changdu Maternity and Child Hospital, Tibet Autonomous Region, Changdu, 854085 China; 4 Obstetrics and Gynecology Department, Fujian Maternity and Child Hospital College of Clinical Medicine for Obstetrics & Gynecology and Pediatrics, Fujian Medical University, Fuzhou, 350001 Fujian Province China

**Keywords:** Incidence, Meta-analysis, Neonatal screening, Primary carnitine deficiency, *SLC22A5*, Variant

## Abstract

**Background:**

Primary carnitine deficiency (PCD) is a rare autosomal recessive fatty acid oxidation disorder caused by variants in *SLC22A5,* with its prevalence and *SLC22A5* gene mutation spectrum varying across races and regions. This study aimed to systematically analyze the incidence of PCD in China and delineate regional differences in the prevalence of PCD and *SLC22A5* gene variants.

**Methods:**

PubMed, Embase, Web of Science, and Chinese databases were searched up to November 2023. Following quality assessment and data extraction, a meta-analysis was performed on screening results for PCD among Chinese newborns.

**Results:**

After reviewing 1,889 articles, 22 studies involving 9,958,380 newborns and 476 PCD cases were included. Of the 476 patients with PCD, 469 underwent genetic diagnosis, revealing 890 variants of 934 alleles of SLC22A5, among which 107 different variants were detected. The meta-analysis showed that the prevalence of PCD in China was 0.05‰ [95%CI, (0.04‰, 0.06‰)] or 1/20 000 [95%CI, (1/16 667, 1/25 000)]. Subgroup analyses revealed a higher incidence in southern China [0.07‰, 95%CI, (0.05‰, 0.08‰)] than in northern China [0.02‰, 95%CI, (0.02‰, 0.03‰)] (*P* < 0.001). Furthermore, the result of the meta-analysis showed that the frequency of the variant with c.1400C > G, c.51C > G, c.760C > T, c.338G > A, and c.428C > T were 45% [95%CI, (34%, 59%)], 26% [95%CI, (22%, 31%)], 14% [95%CI, (10%, 20%)], 6% [95%CI, (4%, 8%)], and 5% [95%CI, (4%, 8%)], respectively. Among the subgroup analyses, the variant frequency of c.1400C > G in southern China [39%, 95%CI, (29%, 53%)] was significantly lower than that in northern China [79‰, 95%CI, (47‰, 135‰)] (*P* < 0.05).

**Conclusions:**

This study systematically analyzed PCD prevalence and identified common *SLC22A5* gene variants in the Chinese population. The findings provide valuable epidemiological insights and guidance for future PCD screening effects in newborns.

**Supplementary Information:**

The online version contains supplementary material available at 10.1186/s13023-024-03267-x.

## Background

Primary carnitine deficiency (PCD, OMIM #212,140) is a rare inherited autosomal recessive disorder of fatty acid oxidation caused by mutations of the solute carrier family 22 member 5 (*SLC22A5*, MIM:603,377) gene. *SLC22A5* encodes the organic cation/carnitine transporter type 2 (OCTN2), which is prominently expressed in the heart, skeletal muscle, kidney, and placenta [1, 2]. Defect in OCTN2 synthesis hinders the reabsorption of carnitine, resulting in low levels of serum carnitine, impairing the transport of long-chain fatty acids from the cytosol to the mitochondria for beta oxidation.

The clinical manifestations of PCD vary widely, ranging from asymptomatic to acute metabolic decompensation early in life, progressive hypertrophic cardiomyopathy, myopathy, and encephalopathy later in life [3–5]. However, untreated patients with PCD may experience sudden death [6], thereby highlighting the importance of timely and continuous carnitine supplementation to prevent metabolic decompensation and ensure favorable long-term outcomes. Hence, early diagnosis is crucial. Newborn screening (NBS) for PCD is performed to measure free carnitine (C0) levels in dried blood spot samples using tandem mass spectrometry (MS/MS). The widespread implementation of NBS for PCD in China has enabled early diagnosis and timely treatment of patients with PCD.

The estimated incidence of PCD varies widely across countries due to region and race, ranging from 1:120,000 to 1:300 newborns [7–10]. Additionally, significant differences in the incidence of PCD have been reported among different regions within China, with rates ranging from 1:100,000 to 1:3000 newborns [11–15]. It is important to clarify the overall prevalence of PCD among the Chinese population, as well as potential regional disparities, notably across the Qinling Mountains-Huaihe River Line that divides China into northern and southern regions. This geographic division introduces variations in the natural environment, geographical landscape, and residents' lifestyles, which may influence disease prevalence. It remains unclear whether there are differences in the prevalence of PCD between the northern and southern regions.

*SLC22A5*, located on chromosome 5q31.1, includes ten exons and three introns and encodes 557 amino acids. Over 180 pathogenic *SLC22A5* gene variants have been identified, and variations have been observed among different racial and regional populations. (http://www.hgmd.cf.ac.uk; data collected on December 15, 2023). Some studies have identified c.51C > G (p.F17L), c.760C > T (p.R254*), and c.1400C > G (p.S467C) as the three most common variants in the Chinese population [16–19]. However, the frequency of the most prevalent variant varies across regions.

To elucidate the epidemiological characteristics of PCD in Chinese populations, a comprehensive meta-analysis was conducted to analyze the nationwide incidence of PCD and clarify the differences in the prevalence of PCD and *SLC22A5* gene variants between northern and southern regions.

## Methods

### Literature search

The systematic review and meta-analysis were conducted according to the Preferred Reporting Items for Systematic Reviews and Meta-Analyses (PRISMA) guidelines [20], with the protocol registered in PROSPERO (ID: CRD42024526722). Three independent researchers (ZJF, LJL, and ZYL) systematically searched databases from 2000 to June 2023 for observational studies on PCD, including English databases encompassing the PubMed, Embase, and Web of Science, and Chinese databases encompassing the China National Knowledge Infrastructure (CNKI), Veipu (VIP), and Wanfang. Search terms comprised (“primary carnitine deficiency” OR “carnitine uptake defect” OR “carnitine transport defect”) AND (“*SLC22A5*” OR “*OCTN2*”) AND (“mutation” OR “variant”) AND (“neonate” OR “newborn” OR “neonate”). Relevant studies on human participants published in English and Chinese were included, and reference lists of relevant reviews and articles were manually examined.

### Eligibility and exclusion criteria

Studies were included if they met the following criteria: (1) original observational studies; (2) studies reporting results of PCD screening for newborns in different provinces, cities, and autonomous regions of China; (3) studies featuring main indicators such as the prevalence of PCD, information on *SLC22A5* gene variants and other relevant PCD-related characteristics, and (4) Studies with relatively high quality.

Studies failing to meet these criteria were excluded from the analysis. In addition, studies with overlapping screening regions or times, low quality, or those not published in English or Chinese languages were excluded.

### Data extraction

Two researchers (ZJF and LJL) extracted the information independently. All relevant data were compiled into a data extraction table based on the specified eligibility and exclusion criteria. Information obtained from the original publications included the first author, publication year, screening year, region, number of NBS participants, number of diagnosed PCD cases, and details on *SLC22A5* gene variants. The frequencies of *SLC22A5* gene variants were calculated from the extraction data. Any discrepancies were resolved through discussion with another investigator (ZYL). Since all analyses relied on previously published studies, ethical approval or patient consent was not required.

### Quality assessment

Two investigators (ZJF and LJL) independently evaluated the quality of included studies using the Agency for Healthcare Research and Quality (AHRQ) criteria. Studies were assigned a score of 1 for compliance with each criterion and a score of 0 for non-compliance or uncertainty, yielding a total score ranging from 0 to 11. Higher scores indicated superior quality, with the included studies categorized as having high, moderate, or low quality, corresponding to scores of 8–11, 4–7, and 0–3 points, respectively. Any discrepancies were thoroughly discussed and resolved by another reviewer (ZYL) if necessary.

### Statistical analyses

All statistical analyses were performed using RevMan version 5.3 (Update Software Ltd., Oxford, Oxon, UK). The chi-square test and *I*^*2*^ were used to evaluate heterogeneity. Heterogeneity was deemed small when the *I*^*2*^ value was less than 50% with a *P* value > 0.10, in which case the fixed-effects model (the Mantel–Haenszel method) was used to estimate the pooled prevalence (OR) and 95% confidence intervals (CIs). Otherwise, a random-effects model (the Der Simonian and Laird method) was employed. Subgroup analysis was conducted to assess the effect of region across the studies. Sensitivity analyses were performed by individually removing studies to evaluate their impact on pooled ORs. Publication bias was visually assessed using a funnel plot and quantitatively evaluated using Begg’s test. A *P* value < 0.1 indicated the existence of publication bias.

## Results

### Study selection

**Fig. 1:**
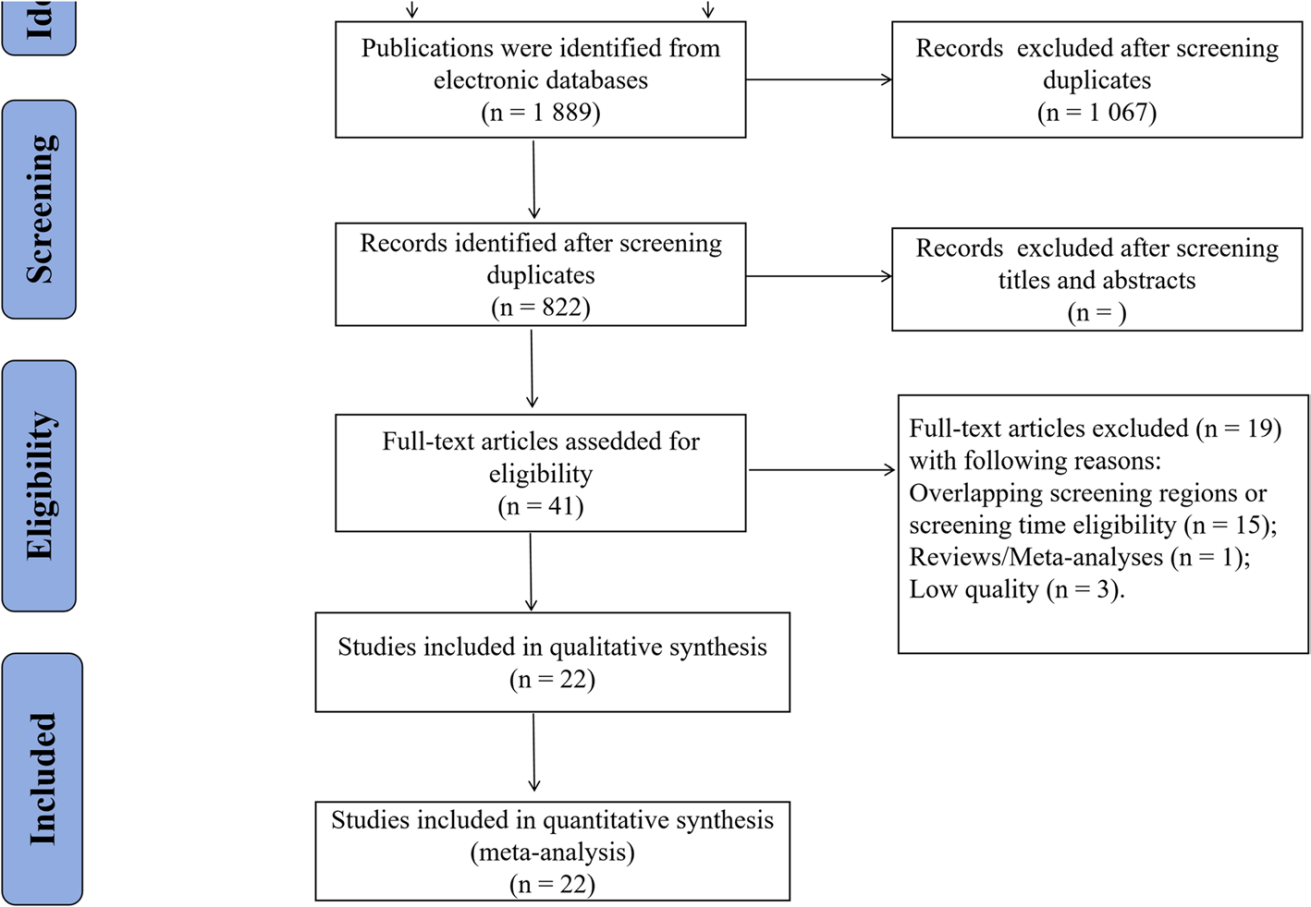
Flow chart of the study selection process

In our initial data search, 1,889 articles (797 in Chinese and 1,092 in English) were identified. Among these, 1,067 articles were excluded after screening duplicates. After reviewing the titles and abstracts of the remaining articles, 781 were further excluded. Subsequently, 41 articles were considered potentially eligible. Upon a thorough review of the full texts, 19 articles were excluded, including those with overlapping screening regions or screening time eligibility (*n* = 15), meta-analyses (*n* = 1), and low-quality studies (*n* = 3). Finally, 30 eligible studies were included in the meta-analysis. A flowchart illustrating the literature search process is shown in Fig. 1.

### Study characteristics

Twenty-two studies involving 9,958,380 newborns were included in our analysis, among which 476 patients were diagnosed with PCD, as shown in Table 1. Notably, 79.53% of the screened newborns resided in southern China. Of the 476 patients with PCD, 469 underwent genetic diagnosis, revealing 890 variants of 934 alleles of *SLC22A5*, among which 107 different variants were detected. The five most prevalent variants accounted for 71.13% of the total number, including c.1400C > G, p.Ser467Cys (29.78%, 265/890), c.51C > G, p.Phe17Leu (19.89%, 177/890), c.760C > T, p.Arg254Ter (14.27%, 127/890), c.428C > T, p.Pro143Leu (4.16%, 37/890), and c.338G > A, p.Cys113Tyr (4.04%, 36/890), as shown in Table 1.

**Table 1:** Characteristics of studies included in the meta-analysis

South or North of China	Reference (author/ year)	Area		Years	Screening Cases	PCD cases	Incidence	No. Of alleles (RF)					AHRQ scores
		Province	City					c.1400C > G (p.S467C)	c.51C > G (p.F17L)	c.760C > T (p.R254X)	c.428C > T (p.P143L)	c.338G > A (p.C113Y)	
South	Chang et al. 2022 [21]		Shanghai	2003–2021	1 247 274	40	1/31 181	17 (17/66, 25.76%)	18 (18/66, 27.27%)	4 (4/66, 6.06%)	2 (2/66, 3.03%)	3 (3/66. 4.54%)	9
	Chen.et al. 2020 [22]	Fujian	Fuzhou	2015–2020	95 453	10	1/9 545	4(4/20, 20.00%)	4(4/20, 20.00%)	4(4/20, 20.00%)	2(2/20, 10.00%)	N/A	9
	Geng et al. 2021 [23]	Guangxi	Nanning	2014–2019	400 575	22	1/18 207	7(7/44, 15.90%)	10(10/44, 22.72%)	2(2/44, 4.54%)	1(1/44, 2.27%)	4(4/44. 9.09%)	8
	Hu et al. 2023 [9]	Anhui	Hefei	2016–2021	631 839	32	1/19 744	29(29/60, 48.33%)	9(9/60, 15.00%)	4(4/60, 6.67%)	3(3/60, 5.00%)	1(1/60, 1.67%)	8
	Huang et al. 2020 [18]	Guangdong	Guangzhou	2015–2019	200 180	15	1/13 345	9(9/30. 30.00%)	6(6/30, 20%)	5(5/30, 16.67%)	1(1/30, 3.33%)	N/A	9
	Li et al. 2022 [24]	Hunan	Changsha	2016–2022	300 849	22	1/13 674	8(8/44, 18.18%)	8(8/44, 18.18%)	6(6/44, 13.64%)	1(1/44, 2.27%)	2(2/44, 4.45%)	9
	Lin et al. 2020 [16]	Zhejiang		2009–2019	3 410 600	113	1/30 182	71(71/226, 31.42%)	33(33/226, 14.60%)	35 (35/226, 15.49%)	9 (9/226, 3.98%)	6 (6/226, 2.65%)	9
	Lin et al. 2021 [17]	Fujian	Quanzhou	2014–2021	548 2 47	49	1/11 188	16 (16/98, 16.33%)	17(17/98, 17.35%)	31 (31/98, 31.63%)	2(2/98, 2.04%)	4 (4/98, 4.08%)	8
	Song et al. 2023 [10]	Fujian	Ningde	2016–2021	148 043	17	1/8 708	9(9/34. 26.47%)	5(5/34, 14.70%)	9(9/34, 26.47%)	1(1/34, 2.94%)	1(1/34, 2.94%)	8
	Tan et al. 2021 [25]	Guangxi	Liuzhou	2016–2020	111 986	12	1/9 332	3(3/24, 12.50%)	8(8/24, 33.33%)	4(4/24, 16.67%)	N/A	3(3/24, 12.50%)	7
	Tu et al. 2023 [26]	Jingxi	Gangzhou	2018–2021	235,644	35	1/6 732	16(16/70, 22.86%)	23(23/70, 32.86%)	3(3/70, 4.28%)	8(8/70, 11.43%)	7(7/70, 10.00%)	8
	Wang et al. 2023 [12]	Hubei	Wuhan	2018–2021	29,948	14	1/2 139	8(8/28, 28.57%)	10(10/28, 35.71%)	3(3/28, 10.71%)	N/A	1(1/28, 3.57%)	5
	Wang et al. 2019 [27]	Jiangsu	Suzhou	2014–2018	401 660	15	1/26 777	15(15/30, 50.00%)	4(4/30, 13.33%)	4(4/30, 13.33%)	1(1/30, 3.335)	N/A	7
	Yang et al. 2021 [14]	Zhejiang	Ningbo	2014–2019	265 524	16	1/16 595	15(15/32, 46.88%)	5(5/32, 15.62%)	2(2/32, 6,25%)	1(1/32, 3.12%)	N/A	8
	Zhou et al. 2022 [28]	Hunan	shaoyang	2016–2020	94 648	5	1/18 929	1(1/10, 10.00%)	2(2/10, 20.005)	2(2/10, 20.00%)	N/A	1(1/10, 10.00%)	8
North	Liu et al. 2023 [29]	Shandong	Rizhao	2016–2022	36 590	1	1/36 590	N/A	N/A	N/A	N/A	N/A	5
	Li et al. 2019 [19]	Henan		2013–2017	720 667	21	1/34 317	16(16/42, 38.10%)	4(4/42, 9.52%)	6(6/42, 14.28%)	2(2/42, 4.76%)	1(1/42, 2.38%)	7
	Tang et al. 2019 [30]	Jiangsu	Lianyungang	2015–2017	110,158	1	1/110158	N/A	N/A	N/A	1(1/2, 50%)	N/A	6
	Wang et al. 2020 [31]		Tianjin	2013–2018	220,443	10	1/20040	5(5/20, 25%)	2(2/20, 10.00%)	1(1/20, 5%)	1(1/20, 5%)	1(1/20, 5%)	9
	Yang et al. 2021 [11]	Shandong	Jining	2014–2019	608,818	16	1/38051	14(14/32, 43.75%)	6(6/32, 18.75%)	N/A	1(1/32, 3.12%)	N/A	7
	Zhou et al. 2019 [32]	Jiangsu	Xuzhou	2015–2017	236,368	10	1/23637	14(14/20, 70%)	N/A	N/A	1(1/20, 5.00%)	N/A	8
	Zhu et al. 2020 [15]	Jilin	Jilin	2015–2018	105,437	1	1/105437	N/A	N/A	N/A	N/A	N/A	7

*Abbreviation*: *PCD* Primary carnitine deficiencymale, *RF* relative frequency, *N/A* not available

#### Assessment of quality

The evaluation results of the AHRQ quality assessment items are also presented in Table 1, indicating that articles with scores four or more were classified as having moderate or high quality. The average score of 7.68 indicated minimal risk of bias.

### Results of *meta*-analysis

#### Incidence of PCD

**Fig. 2:**
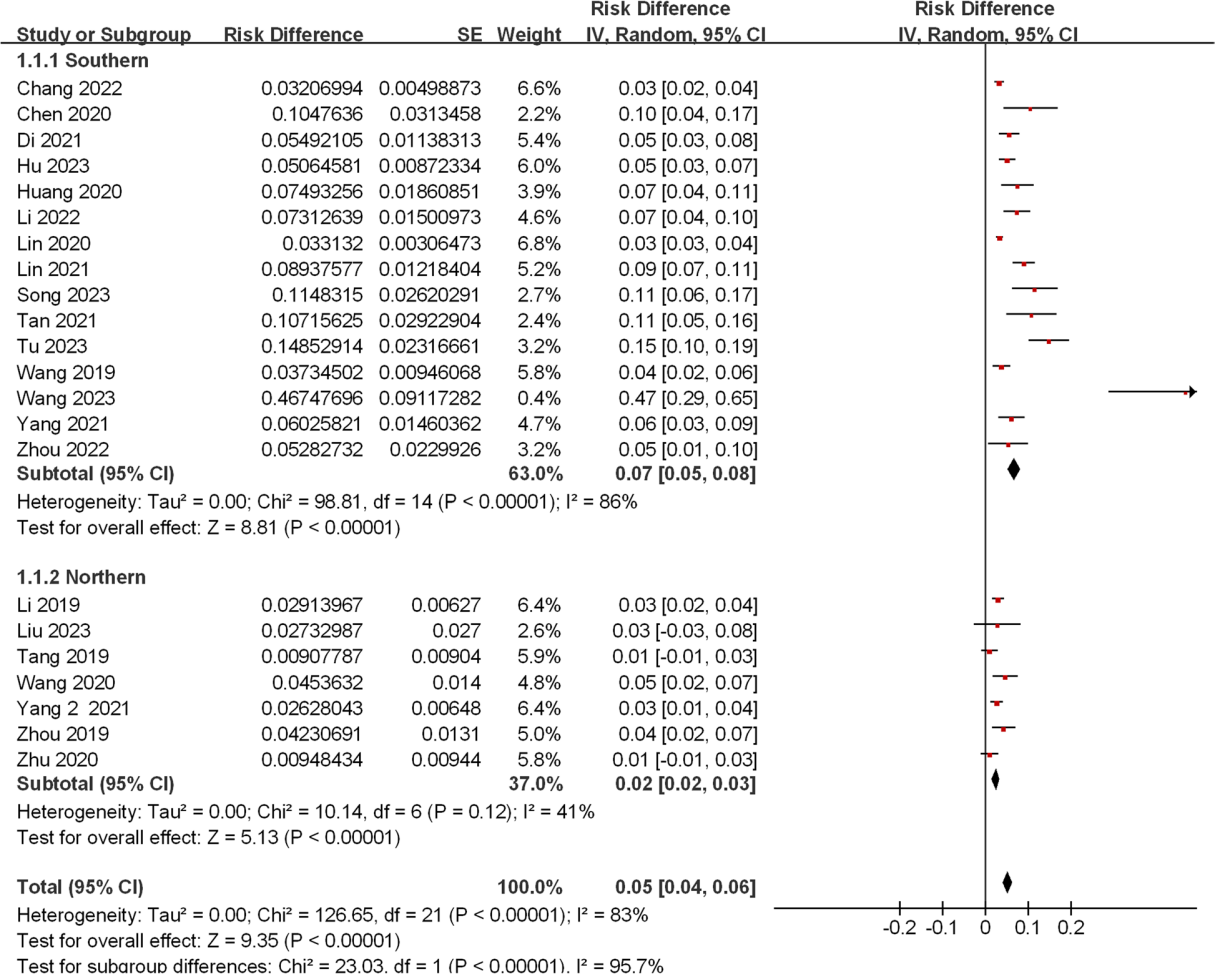
Meta-analysis of the incidence of PCD between southern and northern China

**Fig. 3:**
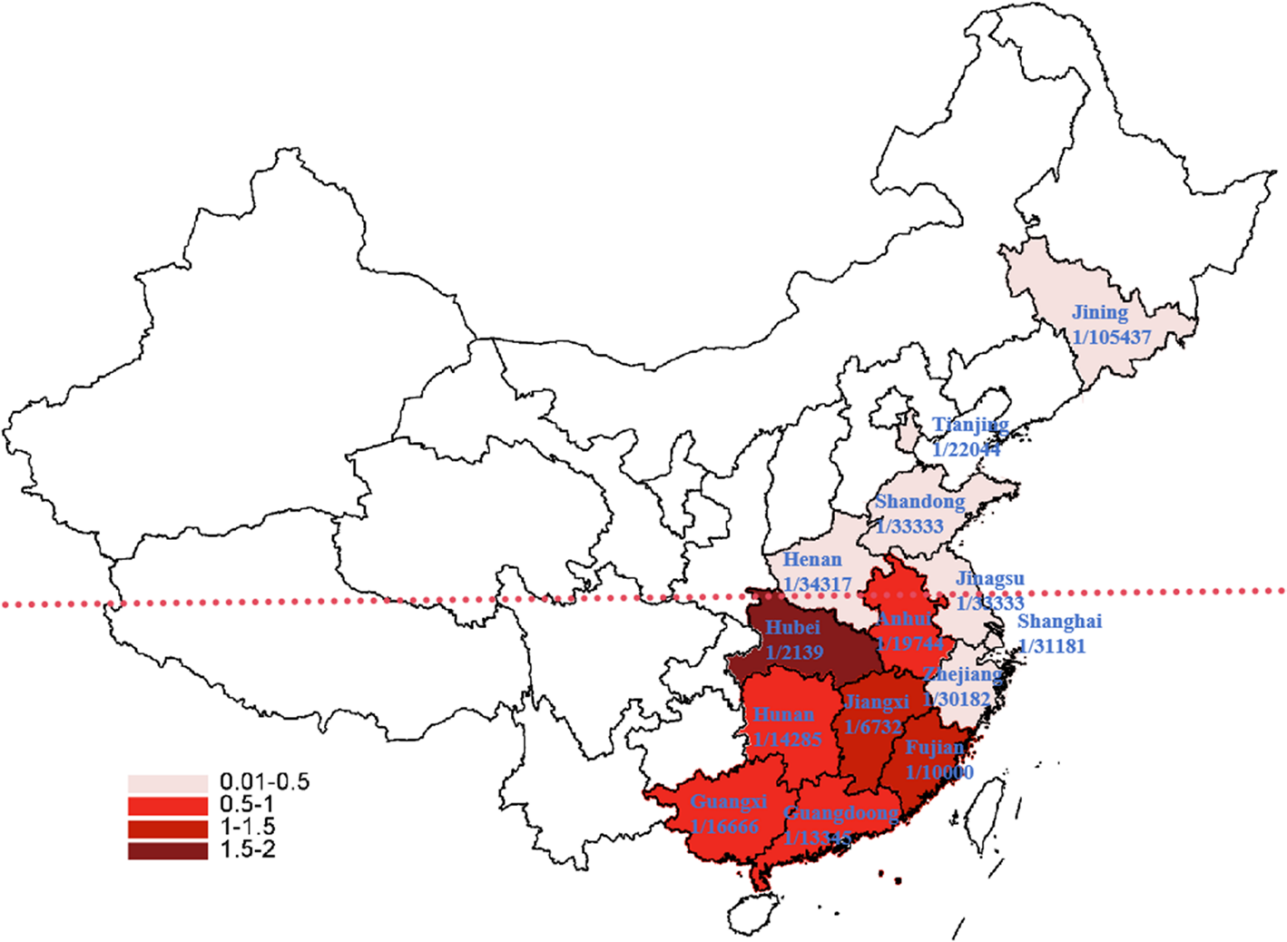
The schematic diagram shows the incidence of PCD in different provinces of China

All included studies reported the incidence of PCD. Due to significant heterogeneity among included studies (*I*^*2*^ = 83%, *P* < 0.05), a random-effects model was employed to analyze the incidence of PCD in China. The meta-analysis showed that the prevalence of PCD was 0.05‰ [95%CI, (0.04‰, 0.06‰)] or 1/20 000 [95%CI, (1/16 667, 1/25 000)] in China (Fig. 2). Subgroup analyses of regional incidence revealed that the incidence in southern China [0.07‰, 95%CI, (0.05‰, 0.08‰)] was greatly higher than that in north China [0.02‰, 95%CI, (0.02‰, 0.03‰)] (*P* < 0.001), as shown in Figs. 2 and 3.

#### Frequency of SLC22A5 gene variants

**Fig. 4:**
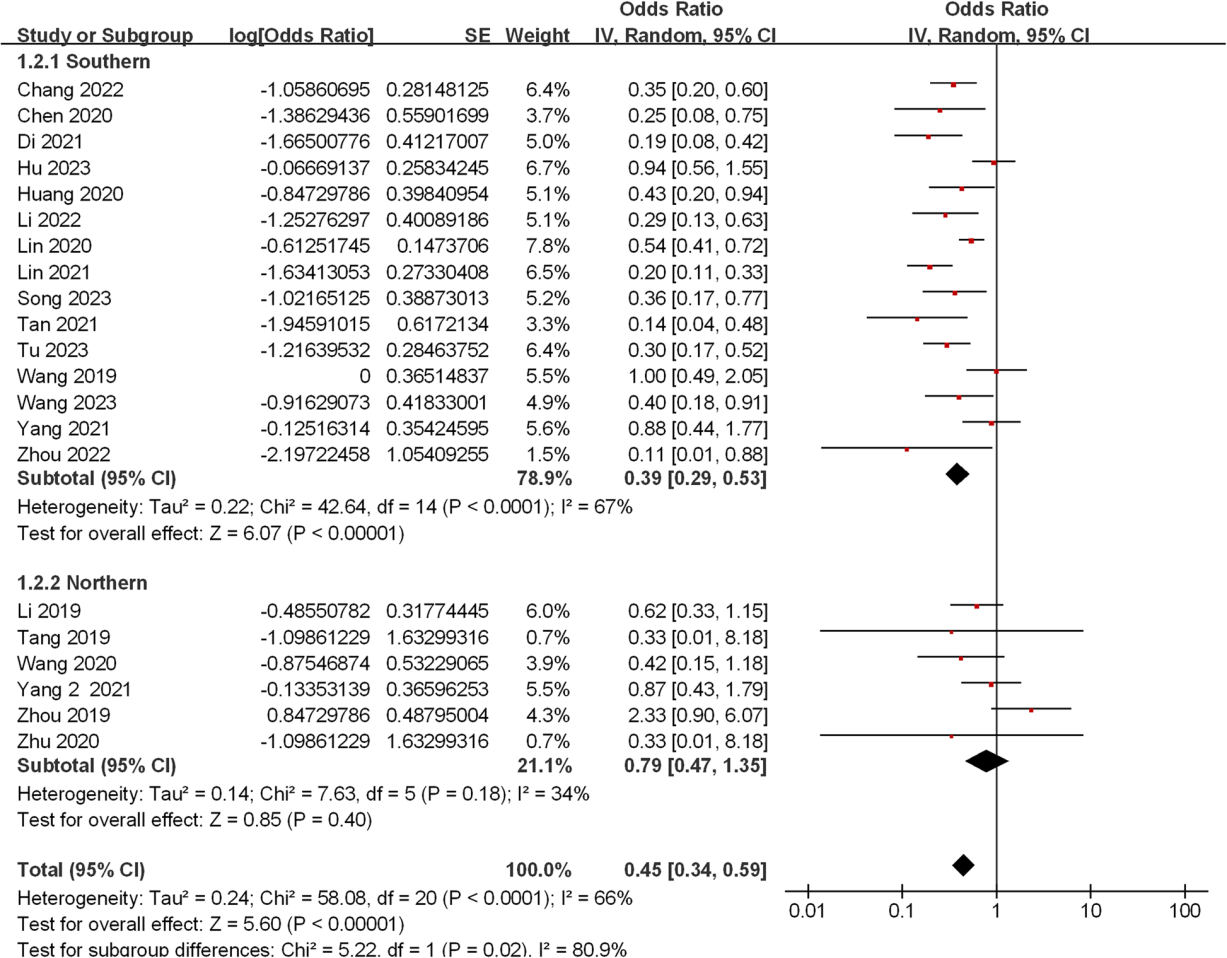
Meta-analysis of the frequency of the c.1400C > G variant of the *SLC22A5* gene between southern and northern China

Among the included studies, 21 reported variant spectra of *SLC22A5* in patients with PCD. We performed a meta-analysis of the frequencies of the five most prevalent gene variants. Since significant heterogeneity was observed among the variant frequencies of c.1400C > G and c.760C > T (*I*^*2*^ = 66% and 76%, *P* < 0.05), a random-effects model was used for the analysis. No significant heterogeneity was identified among the variant frequencies of c.51C > G, c.428C > T, and c.338G > A (*I*^*2*^ = 29%, 0%, and 0%, *P* > 0.05), and a fixed-effects model was used for the analysis. The result of the meta-analysis showed that the frequency of the variant with c.1400C > G, c.51C > G, c.760C > T, c.338G > A, and c.428C > T were 45% [95%CI, (34%, 59%)], 26% [95%CI, (22%, 31%)], 14% [95%CI, (10%, 20%)], 6% [95%CI, (4%, 8%)], and 5% [95%CI, (4%, 8%)], respectively. Among the subgroup analyses, the variant frequency of c.1400C > G in southern China [39%, 95%CI, (29%, 53%)] was significantly lower than that in northern China [79‰, 95%CI, (47‰, 135‰)] (*P* < 0.05) (Fig. 4), while there was no statistically significant difference in the frequency of other variants between southern and northern China (Fig. S1-4).

#### Publication bias analysis

**Fig. 5:**
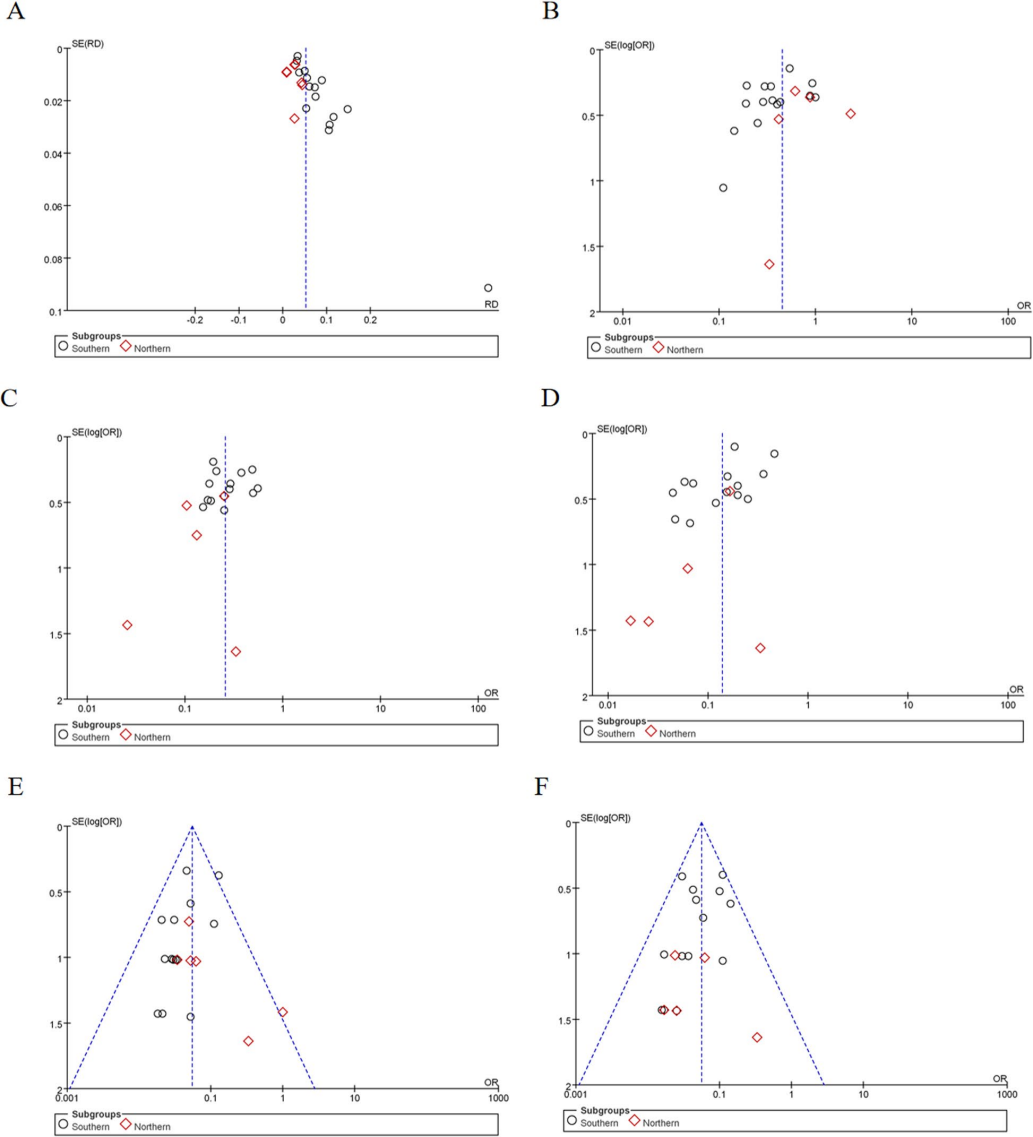
Funnel plots for publication bias. **A** Funnel plot of the incidence of PCD. **B** Funnel plot of the frequency of the c.1400C > G variant of the *SLC22A5* gene. **C** Funnel plot of the frequency of the c.51C > G variant of the *SLC22A5* gene. **D** Funnel plot of the frequency of the c.760C > T variant of the *SLC22A5* gene. **E** Funnel plot of the frequency of the c.428C > T variant of the *SLC22A5* gene. **F** Funnel plot of the frequency of the c.338G > A variant of *SLC22A5* gene

Funnel plots were utilized to evaluate publication bias for the incidence of PCD and the frequency of *SLC22A5* gene variants. The plots were approximately symmetrically distributed, indicating no significant publication bias (Fig. 5).

## Discussion

Our meta-analysis included 22 studies on PCD screening for newborns conducted in 12 provinces (municipalities) across China over the past two decades, including approximately 10 million newborns. This analysis is currently the most comprehensive and systematic review of PCD screening worldwide. Our findings revealed a prevalence of PCD of approximately 1 in 20,000 Chinese newborns, with a significantly higher prevalence noted in southern China than in northern China. Benefiting from the extensive sample size and the relative representativeness of regional population distribution, along with the absence of publication bias in the literature, the results of our meta-analysis offer an objective and reliable assessment.

Timely detection, diagnosis, and intervention for PCD through NBS are crucial to mitigate severe clinical outcomes in affected individuals [21–23]. With the widespread application of MS/MS and genetic testing, PCD can be promptly diagnosed and managed.

Globally, the Faroe Islands exhibit the highest incidence of PCD, with a prevalence of up to 1:297 [7]. This ratio varies across different regions, estimated at 1:50,000 in the United States [9], 1:25,000 in Egypt [24], 1:30,000 in Thailand [10], and 1:40,000 in Japan [25]. It is worth noting that the estimated prevalence of PCD is 1:17,641 in China based on the carrier frequency of SLC22A5 pathogenic or likely pathogenic (P/LP) variants from the Chinese Newborn Genome Project [26]. Additionally, a national cross-sectional survey included 7 million newborns and reported a prevalence of 1:20,284 in mainland China [27], consistent with our study findings. Subgroup analysis revealed a significantly higher prevalence of PCD in southern China, particularly in the Fujian [17, 28, 29], Jiangxi [30], and Guangxi Provinces [31, 32], indicating a geographical trend with higher prevalence in southern China and lower prevalence in northern China.

Uncovering the mutation spectrums of *SLC22A5 gene* plays an important role in clarifying the correlation between genotype and phenotype, genetic counseling, and management of PCD. Several ethnic-specific variant spectra of *SLC22A5* have been identified in different populations. For example, c.95A > G (p.N32S) is predominant in the Faroe Islands [7], c.136C > T (p.P46S) in the United States [33], c.51C > G (p.Phe17Leu) in Thailand [10], c.760C > T (p.R254X) and c.454G > C (p.G152R) in Turkey [34], and c.1400C > G (p.S467C) in Japan [25].

Our analysis revealed that the top five mutations collectively accounted for 71.13% of the total number, providing convincing evidence for the rapid detection of targeted variants of the *SLC22A5* gene in the Chinese population. While studies have consistently identified c.1400C > G (p.S467C), c.51C > G (p.F17L), and c.760C > T (p.R254*) as the top three prevalent variants in the Chinese population, the variant with the highest frequency varies across different geographic regions. Subgroup analysis indicated that the c.760C > T mutation, which results in very low residual OCTN2 transporter activity and obvious clinical manifestations, was most frequent in the Fujian Province [17], while c.1400C > G, with residual OCTN2 transporter activity that may lead to mild phenotypes, predominated in Jiangsu [35], Shandong [11], and Henan [19] Provinces. In contrast, c.51C > G was the most prevalent variant in Shanghai [36] and Guangxi [30–32] Provinces. Our findings underscore regional disparities, with c.1400C > G exhibiting a higher frequency in northern China than in southern China.

## Conclusions

Our systematic review and meta-analysis of NBS results for PCD in China yielded a comparatively accurate prevalence of 1/20 000. Our findings highlight a significantly higher incidence of PCD in southern China than in northern China. Additionally, we confirmed the three most common variants of *SLC22A5* in the Chinese population, noting a higher frequency of c. 1400C > G variant in northern China than in southern China. However, the coverage rate of MS/MS-based newborn screening, being a voluntary chargeable program in China, remains relatively low in northwest China due to poor medical and economic conditions. Consequently, the number of included studies in northern China is relatively limited compared to that in southern China. Overall, our study provides valuable epidemiological insights into PCD in the Chinese population, guiding future endeavors in NBS for PCD.

### Supplementary Information

Supplementary Material 1: Fig. S1. Meta-analysis of the frequency of the c.51C>G variant of the*SLC22A5* gene between southern and northern China.

Supplementary Material 2: Fig. S2. Meta-analysis of the frequency of the c.760C >T variant of the*SLC22A5* gene between southern and northern China.

Supplementary Material 3: Fig. S3. Meta-analysis of the frequency of the c.428C>T variant of the*SLC22A5* gene between southern and northern China.

Supplementary  Material 4: Fig. S4. Meta-analysis of the frequency of the c.338G>A variant of the*SLC22A5* gene between southern and northern China.

## Data Availability

All data generated or analyzed during this study are included in the article; further inquiries can be directed to the corresponding author.

## Abbreviations

PCD: Primary carnitine deficiency
NBS: Newborn screening
OCTN2: Organic cation/carnitine transporter type 2
C0: Free carnitine
MS/MS: Tandem mass spectrometry
