# Supplementary figures and images for: Screening primary carnitine deficiency in 10 million Chinese newborns: a systematic review and meta-analysis
Source: Orphanet J Rare Dis. 2024 Jul 3;19:248. doi: 10.1186/s13023-024-03267-x (PMC11220949; doi:10.1186/s13023-024-03267-x)

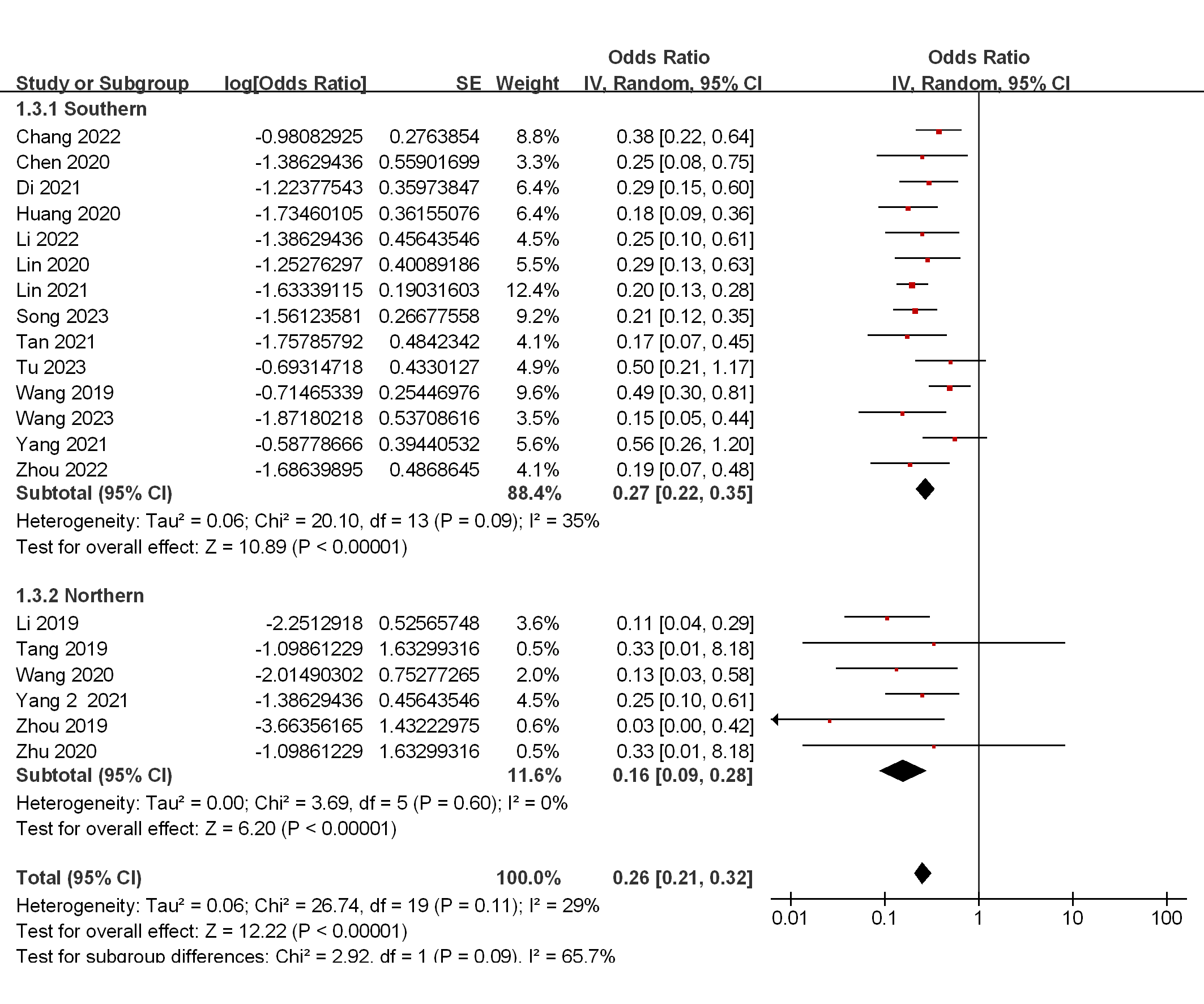

Supplement: Supplementary file 1 — Supplementary Material 1: Fig. S1. Meta-analysis of the frequency of the c.51C>G variant of theSLC22A5 gene between southern and northern China. [file 13023_2024_3267_MOESM1_ESM.tif]

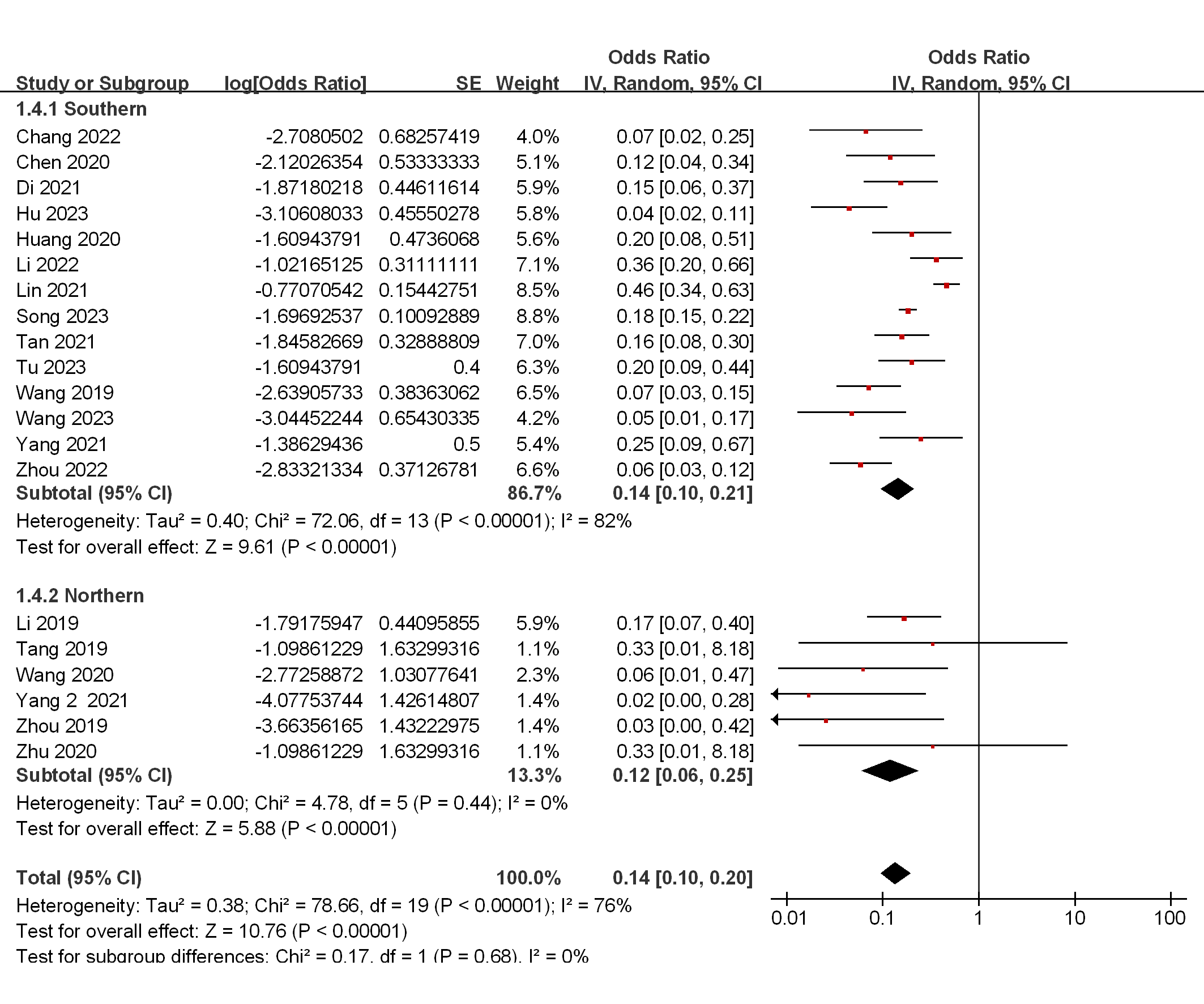

Supplement: Supplementary file 2 — Supplementary Material 2: Fig. S2. Meta-analysis of the frequency of the c.760C >T variant of theSLC22A5 gene between southern and northern China. [file 13023_2024_3267_MOESM2_ESM.tif]

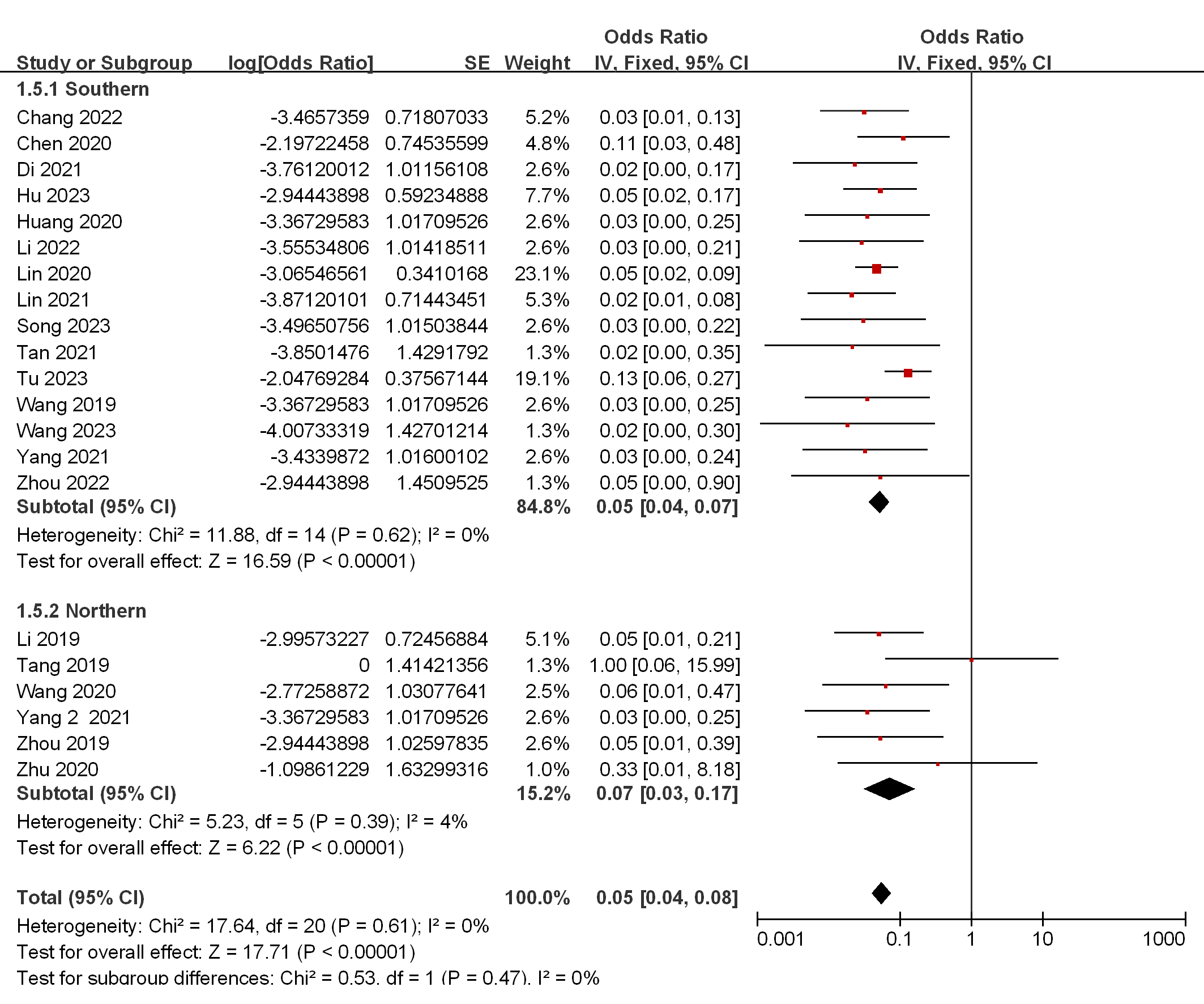

Supplement: Supplementary file 3 — Supplementary Material 3: Fig. S3. Meta-analysis of the frequency of the c.428C>T variant of theSLC22A5 gene between southern and northern China. [file 13023_2024_3267_MOESM3_ESM.tif]

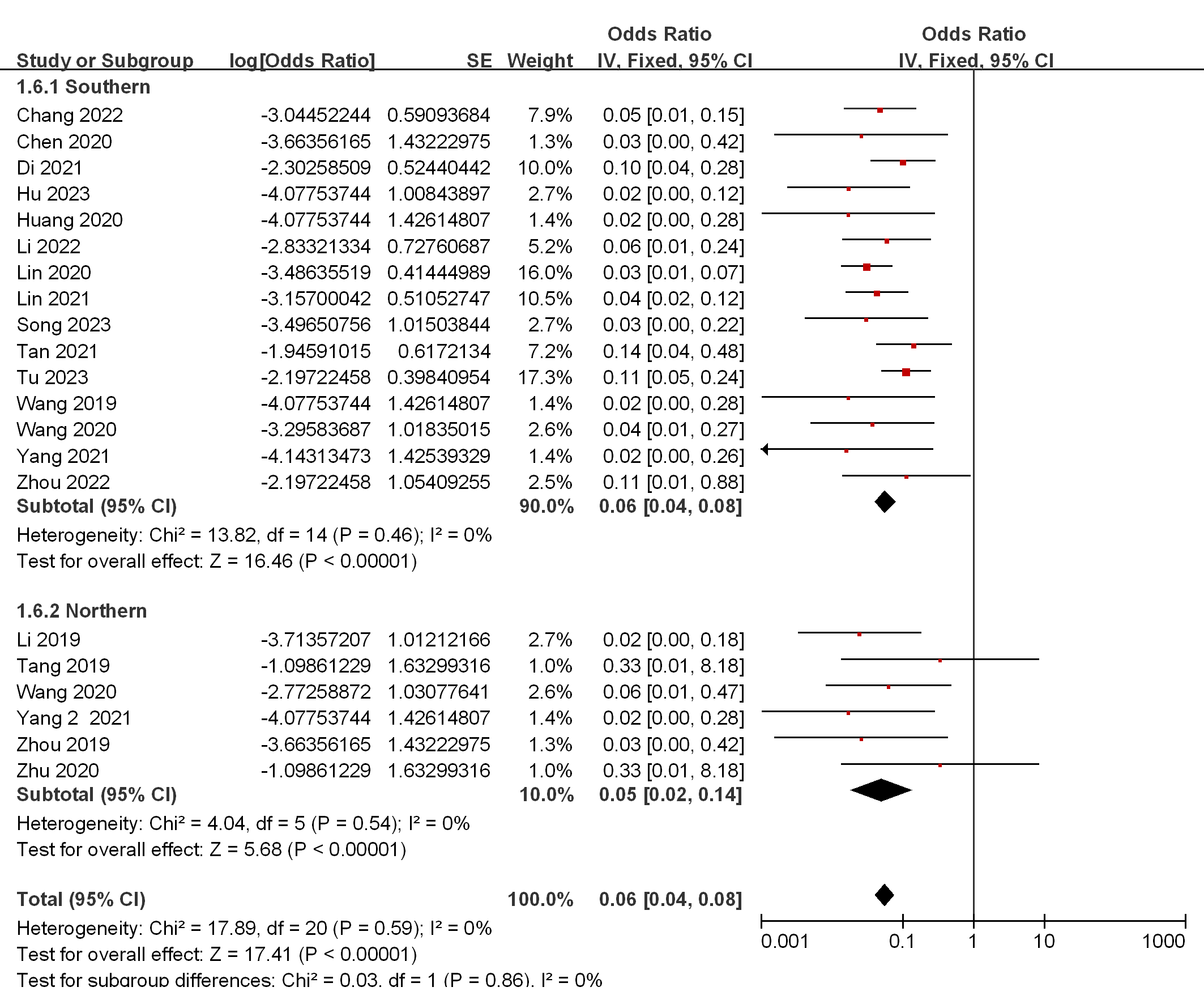

Supplement: Supplementary file 4 — Supplementary Material 4: Fig. S4. Meta-analysis of the frequency of the c.338G>A variant of theSLC22A5 gene between southern and northern China. [file 13023_2024_3267_MOESM4_ESM.tif]
